# Detection of anti-HEV antibodies but no molecular positivity in dogs in the Campania region, southern Italy

**DOI:** 10.1016/j.onehlt.2024.100724

**Published:** 2024-04-08

**Authors:** G. Ferrara, U. Pagnini, E. Improda, R. Ciarcia, A. Parisi, F. Fiorito, G. Della Valle, G. Iovane, S. Montagnaro

**Affiliations:** Department of Veterinary Medicine and Animal Productions, University of Naples Federico II, Via Federico Delpino n.1, 80137 Naples, Italy

**Keywords:** Hepatitis E virus, HEV, Paslahepevirus balayani, Stray dogs, One health

## Abstract

Paslahepevirus balayani genotypes 3 and 4 (HEV-3 and 4) have zoonotic potential and can be transmitted to humans and animals through the consumption of contaminated raw or undercooked meat. Although it has been demonstrated that dogs are susceptible to the infection and produce specific antibodies, the epidemiological role of this species is not yet well defined. This study aimed to evaluate the circulation of HEV at the serological and molecular level in the dog population of the Campania region, southern Italy. A total of 231 dogs were sampled, divided according to several variables (sex, age, origin, lifestyle, location, size, and breed), and tested for the presence of HEV antibodies using a commercial multi-species ELISA. A total of 197 blood samples and 170 stool samples were tested with two specific PCRs in order to detect viral RNA. A total of 19 out samples of 231 were seropositive, obtaining an exposure (8.2%) similar to that observed in other European countries. The univariate and multivariate analysis revealed a wide exposure to stray dogs and animals from the province of Salerno. All samples tested with molecular methods were negative. Defining the role of domestic carnivores continues to be a “one health” challenge, although it appears that they do not eliminate the virus and therefore do not pose a danger to humans. In the absence of other evidence, it is advisable to continue to carry out surveillance also for domestic animals, which, due to ethological characteristics or their position in the food chain, could be predisposed to being exposed to HEV.

## Highlights

• Dogs in southern Italy were exposed to HEV.
• A higher seroprevalence was found in stray dogs and in the Salerno province.
• Although a large-scale molecular investigation was carried out, no sample was found to be positive.

## 1 Introduction

The taxonomy relating to the hepatitis E virus (HEV) has recently (from 2021) undergone changes, and now the genera Paslahepevirus, Avihepevirus, Rocahepevirus, and Chirohepevirus are described in the Hepeviridae family [1,2]. The genus Paslahepevirus contains Paslahepevirus balayani (formerly known as Orthohepevirus A, a positive-sense, single-stranded RNA virus with three open reading frames) and Paslahepevirus alci. Paslahepevirus balayani comprises eight genotypes, including those infecting humans exclusively (1 and 2) and those with zoonotic potential (3 and 4) [1,2]. HEV-1 and 2 are spread through the orofecal route (contaminated food or drinking water) and are particularly prevalent in developing African and Asian countries. Pigs and wild boars are the reservoirs of HEV-3 and HEV-4, which can be transmitted to several species, including humans [1]. Unlike genotypes 1 and 2, genotypes 3 and 4 are transmitted, in both animals and humans, by the consumption of contaminated raw or undercooked meat and are responsible for outbreaks in industrialized countries. Although HEV is considered under-recognized, the WHO estimates 20 million infections globally, accounting for 5–15% of all acute hepatitis infections of unknown origin in Europe.

Although these infections are completely asymptomatic in animals and do not cause any tissue damage, the infection can cause severe chronic hepatitis in humans, especially in immunocompromised patients (the mortality is estimated at around 0.5%) [1]. The involvement of other mammals in the viral cycle complicates this relatively simple epidemiological scenario. While the role of lagomorphs as secondary reservoirs of infection (in particular by hares) is clear, the role of carnivores is still debated due to their “undetectable” infection [3,4]. Over the years, there have been reports of serological positivity in domestic carnivores in the Western world, in some cases even with high prevalences that suggested intense virus circulation or exposure. However, all attempts to detect the virus in blood, feces or liver tissue have failed [5,6]. The situation is different among wild carnivores, where molecular positivity has occasionally been reported in addition to significant serological prevalences [7]. All these aspects of the complex epidemiological cycle of HEV have been highlighted in Italy [8]. In this country, the virus is occasionally described in humans, while it is widespread in pig farms and among wild boars [9,10]. Reports of viral circulation among wild and domestic animals, including dogs, are regularly reported, although a study involving consistent sampling has never been conducted for this species. Due to the uncertainty that HEV can be transmitted to humans, the observation of HEV exposure in domestic animals in close contact with humans provides a possible zoonotic threat, especially considering the recent increase of stray dogs and wildlife populations. Considering this context, the current investigation was intended to assess the presence of anti-HEV antibodies as well as viral RNA in dogs sampled from the Campania region, southern Italy.

## 2 Materials and methods

### 2.1 Sampling and study area

This study examined blood serum samples from 231 dogs from the Campania region, Southern Italy (40°49′34″N 14°15′23″E). Campania is a region with 5,597,358 inhabitants and is the most populous and densely populated region in the South. Located between the Tyrrhenian Sea in the south-west and the southern Apennines in the north-east and has an area of 13,670.95km^2^. It is also one of the regions with the largest dog population. Given the abundance of game animals, the practice of hunting is widespread in this region (especially wild boar hunting), and therefore the breeding of hunting dogs is also a common practice [11]. The sample size was calculated using the formula proposed by Thurshfield for a theoretically “infinite” population and add the following information: expected prevalence of HEV (10%), 95% confidence interval (CI) and desired absolute precision (5%) [12]. In particular, a non-proportional stratified random sample was used in which a dog population was divided into different strata (stray dogs, hunting dogs, companion dogs) with respect to the five provinces of the Campania region (Naples, Salerno, Benevento, Avellino and Caserta). A random sample was drawn within each stratum. A total of 52 hunting dogs, 117 stray dogs, and 62 pet dogs comprised the sampling. A whole blood sample (for serum) and a blood sample with an anticoagulant (for RNA extraction) were collected from 197 dogs. Fresh fecal samples from 170 animals were also collected. All samples were transported under cold chain conditions to the Department of Veterinary Medicine and Animal production of Naples. There, each sample was processed (e.g. blood was centrifuged to obtain serum samples) and/or stored at −80 °C before being tested (serum samples for serology were stored at −20 °C). Information on each sampled animal (sex, breed, size, province, age, origin, and lifestyle) was collected using a questionnaire. The animal study protocol was approved by the Institutional Ethics Committee of Department of Veterinary Medicine and Animal Production (Centro Servizi Veterinari), University of Naples, Federico II (PG/2022/0093420, 21st July 2022).

### 2.2 Serological assay and statistical analysis

According to the manufacturer's instructions, a commercial multispecies ELISA kit (Dia.Pro, Diagnostic Bioprobes Srl) was used to identify specific antibodies against HEV [13]. Briefly, 100 μl of negative control, positive control, and samples were incubated for 45 min at 37 °C in the wells of 96-multiwells (coated with highly specific synthetic antigen encoding for conservative and immunodominant determinants of HEV). After three washing steps, 100 μl of conjugate (synthetic antigen labeled with peroxidase) were added and incubated as previously described. Further three washing steps preceded the addition of the substrate solution (3,3′,5,5′-tetramethylbenzidine) and, after twenty minutes at room temperature, the supplementation of the stop solution (0.2 M H_2_SO_4_). The optical density (OD) of each sample was measured using a spectrophotometer. A cut-off value was calculated according to the manufacturer's instructions in order to classify the sample as positive or negative.

The chi-square statistics was used to evaluate the relationship between dependent (ELISA outcome) and independent variables. The independent variables were sex (male or female), age (≤2 years considered young, >2 and ≤ 6 years considered adult, <6 years considered old), province (Avellino, Benevento, Salerno, Caserta, Napoli), life-style (hunting and non-hunting), breed (mix or pure breed), origin (owned or stray), and size (≤ 15 kg considered small, >15 and ≤ 25 considered medium and > 25 kg considered giant). A *p*-value <0.05 was considered significant. All significant variables for univariate analysis were assessed using the forward elimination strategy in a logistic regression. The degree of correlation between independent factors and HEV seropositivity was calculated using odds ratios (OR) and 95% confidence intervals. The Akaike Information Criterion (AIC) was used to assess fit models, and those that best matched the data were selected. The Variance inflation factor (VIF) was used to assess collinearity. MedCalc Statistical Software version 16.4.3 (MedCalc Software, Ostend, Belgium) and JMP version 14.1.0 (SAS Institute Inc.) were used for the statistical analysis.

### 2.3 RT real time PCR

Viral RNA extraction from blood and feces samples was performed using the QIAamp Viral RNA Mini Kit (Qiagen) and following the manufacturer's instructions. Each RNA was quantified using a nanodrop and retrotranscribed using a commercial kit (iScript™ cDNA Synthesis Kit, Bio-Rad) [12]. The obtained cDNAs were used as templates for two different PCR protocols previously described in the literature that succeeded in the detection of HEV RNA in similar samples. The first was a real-time PCR performed using iTaq Universal Probes Supermix (Bio-Rad) employing the following primers and probes: F 5′-RGTRGTTTCTGGGGTGAC-3′; R 5′-AKGGRTTGGTTGGRTGA-3′; probe 5′-FAM-TGAYTCYCARCCCTTCGC-TAMRA-3′. The thermal conditions included an initial denaturation of 15 min at 95 °C, followed by 40 cycles of denaturation at 95 °C (10 s), annealing at 51 °C (30 s), and extension at 60 °C (20 s) [2,7]. Results were read using a CFX96™ Real-Time PCR Detection System (Bio-rad). The second amplification attempt was performed through a nested PCR using Taq DNA Polymerase (Qiagen), forward primer (5’-ACYTTYTGTGCYYTITTTGGTCCITGGTT-3′) and reverse primer (5′- GCCATGTTCCAGAYGGTGTTCCA-3′) [14]. The amplification products were run on agarose gels electrophoresis and observed with a transilluminator. Positive samples from previous studies were used as positive controls in the different PCR protocols.

## 3 Results

An overall seroprevalence of 8.2% was observed (anti-HEV antibodies were detected in 19 serum samples). Risk factors analysis revealed no differences in HEV exposure based on sex, breed, or size (Table 1). Surprisingly, no differences were observed between dogs used for hunting and dogs with different lifestyle (*p* = 0.87) although higher seropositivity was found in non-hunting dogs (8.4%). Higher seroprevalences, although not significant, were obtained in older animals (11.6%) compared to adult or young animals (11.1%). Most of the seropositive animals came from the province of Salerno, which has a higher seroprevalence than other provinces. Likewise, stray origins were statistically correlated with a higher risk of HEV exposure. The multivariate analysis of risk factors (Table 2) identified the correlation between location (province of Salerno) and higher seroprevalences (OR = 10.3).

**Table 1:** HEV seroprevalence in the dog population of the Campania region. **Description:** Univariate analysis (chi-square) of potential risk factors (province, sex, age, bred, origin, size, and attitude) for HEV seropositivity.

	HEV					
Factor	n	Positive	%	95%CI	χ^2^	p
Total	231	19	8.2	4.7–11.8		
Province						
Avellino	34	1	2.9	0.0–8.6		
Benevento	28	1	3.5	0.0–10.5		
Salerno	52	11	21.1	10.1–32.3	15.1	**0.004**
Caserta	65	3	4.6	0.0–9.7		
Napoli	52	3	5.8	0.0–12.1		
Sex						
Male	136	8	5.9	2.1–10.6		
					2.4	0.12
Female	95	11	11.6	2.8–14.0		
Age						
Young	54	3	5.5	0.0–11.7		
Adult	105	8	7.6	2.5–12.7	1.35	0.5
Old	72	8	11.1	3.8–18.4		
Bred						
Mix	125	12	9.6	4.4–14.8		
					0.68	0.4
Specific bred	106	7	6.6	1.9–11.3		
Origin						
Stray	139	16	11.5	6.2–16.8		
					4.9	**0.025**
Owned	92	3	3.3	0.0–6.9		
Size						
Small	77	9	11.7	4.5–18.9		
Medium	106	6	5.7	1.3–10.1	2.1	0.34
Giant	47	4	8.5	0.5–16.5		
Life-style						
Hunting	52	4	7.7	0.5–14.9		
					0.02	0.87
Non-hunting	179	15	8.4	4.3–12.4		

n = number.

χ^2^ = chi square.

CI = confidence interval.

**Table 2:** Multivariate risk factor analysis. **Description:** Multivariate risk factor analysis and calculation of odds ratio (OR).

Factor	Coefficient (β)	OR	95% CI	p-value
Origin (Stray)	1.25	0.17	0.9–13.5	0.07
Province (Benevento)	−0.014	0.99	0.06–16.6	0.99
Province (Caserta)	0.58	1.78	1.24–85.45	0.62
Province (Salerno)	2.33	10.3	2.7–76.6	**0.03**

OR = Odds ratio.

CI = confidence interval.

No sample (neither blood nor feces) tested positive for the two nucleic acid amplification methods used (real-time and nested PCR).

## 4 Discussion

Serological evidence of exposure to HEV had already been described in hunting dogs in Italy, although in small-scale studies that had described 14.35% and 5% of seropositive animals, in 2015 and 2022 respectively (in the latter case, molecular investigations on blood samples and laboratory analyses aimed at evaluating signs of liver failure were inconclusive) [13,15]. The seroprevalence found in our study (8.2%) can be considered similar to those obtained in studies carried out in other European countries. For example, in Spain, 9.9% of tested dogs revealed antibodies against HEV (whose specificity was further confirmed by western blot analysis, although RNA was not detected in any of the tested serum samples) [6]. A study performed in the Netherlands found HEV-specific antibodies in 18.52% of pet dogs but viral RNA was not detected in these animals [16]. Similarly, a seroprevalence of 21.1% was found in Bulgaria [17]. Even in Germany and Switzerland, where >50% and 38% of the animals showed antibodies against HEV, respectively, the virus was not detectable [18,19]. Other studies have highlighted prevalences of 10% in Germany, 22.7% in India, and 6.97% in Brazil [[20], [21], [22]]. Accordingly, research conducted in the UK obtained a serological prevalence of 2.2% but no positive stool samples, testing 248 animals by real-time PCR. The same study also looked for viral RNA in 84 liver samples, but with the same negative outcome [23]. Nevertheless, studies conducted in Asian regions reveal higher prevalences. Seroprevalences of 21.12% and 28.2% have been reported in China and South Korea, respectively [24,25]. The only studies that have identified risk variables associated with increased HEV exposure in hunting dogs were conducted in China, with significantly higher prevalences in older dogs and stray dogs [24]. The discrepancies observed among all these studies are related not only to real differences in the circulation of the infection among domestic animals, but also to intrinsic investigation variables such as the type and amount of sample and the test used [26]. Nonetheless, our findings support a previously recognized trend in the literature: despite the fact that dogs are known to be exposed to HEV, it is not possible to identify molecularly positive regardless of which matrix is analyzed. Although this trend is typical among pets (dogs and cats), the same cannot be said for wildlife, as evidenced by multiple identifications of viral RNA in different matrices. To date, HEV has been identified in the feces of wolves, red foxes, and lynxes and in the cavity transudates of red foxes [7,[27], [28], [29]]. Furthermore, in wild animals, exposure to the virus appears significantly higher, probably due to greater contact with reservoir hosts and therefore a greater possibility of contracting the infection through their ingestion. For example, seroprevalences of 18.2% and 53.8% were described, respectively, in Iberian lynxes and raccoons, whereas long-term surveillance (from 1993 to 2012) showed how red fox seropositivity oscillates between 40 and 100% in Germany [7,19,29]. Given that the identification of HEV RNA occurred primarily in studies conducted on wild populations with high seroprevalences and large sample sizes, we can assume that it is due to a probabilistic fact and that the virus could have been identified in domestic animals if the same conditions had been met. However, large-scale studies are described, as well as studies carried out on hunting dogs and stray dogs which, like wild animals, should show a predisposition to contract this infection for a closer contact with reservoirs. In our study, we observed greater exposure in stray dogs (but not in hunting dogs), but, despite this, the viral RNA was not identified even in these animals.

The most important question is: do pets become infected, and are they able to eliminate the virus? Should they be considered components of the epidemiological cycle and therefore need to be monitored for the control of infection in humans? There are not yet such certainties and evidence in the literature that would allow pets to be excluded from the epidemiological cycle of HEV (in the absence of evidence supported by experimental infection). Furthermore, several sequenced HEV strains found in wild animals revealed high degrees of nucleotide homology towards rodent-related HEV groups [7]. This aspect indicates that even positive findings in wild animals are related to the transit of contaminated food (e.g., rats) rather than real viral reproduction and elimination. Assuming this theory is correct, dogs will be considered nothing more than mechanical and passive carriers of the virus.

There are several examples of investigations conducted in Italy on populations other than dogs. As an emerging disease with a high rate of spread, numerous species exhibit receptivity, such as wild boars, domestic pigs, red foxes, cats, and crested porcupine [[30], [31], [32], [33], [34]]. All of this evidence emphasizes the spread of HEV in Italy and the importance of a monitoring system that focuses workers at occupational hazard (veterinarians, farmers, hunters, etc.) [22,35].

## 5 Conclusions

This study highlighted the exposure of the canine population of the Campania region, Italy, to HEV, although it failed to demonstrate the presence of viral RNA in blood and fecal samples from the same animals. Stray animals exhibited higher levels of exposure, most likely due to easier contact with reservoir hosts. The role of domestic carnivores in HEV epidemiology is uncertain. In the lack of other evidence, it is prudent to monitor the virus's spread in all susceptible species as if they were all shedders.

## Funding

The authors did not receive any financial support for this work.

## CRediT authorship contribution statement

**G. Ferrara:** Conceptualization, Data curation, Investigation, Methodology, Resources, Validation, Writing – original draft, Writing – review & editing. **U. Pagnini:** Conceptualization, Supervision, Validation, Writing – review & editing. **E. Improda:** Investigation, Resources. **R. Ciarcia:** Investigation, Resources. **A. Parisi:** Methodology, Resources. **F. Fiorito**: Investigation, Resources. **G. Della Valle:** Investigation, Resources. **G. Iovane:** Data curation, Validation, Visualization. **S. Montagnaro:** Supervision, Validation, Visualization, Writing – review & editing.

## Declaration of competing interest

All the authors declare that there's no financial/personal interest or belief that could affect the objectivity of this article.

## Data Availability

All data generated or analyzed during this study are included in this published article.
